# Label-Free Detection of African Swine Fever and Classical Swine Fever in the Point-of-Care Setting Using Photonic Integrated Circuits Integrated in a Microfluidic Device

**DOI:** 10.3390/pathogens13050415

**Published:** 2024-05-16

**Authors:** Georgios Manessis, Maciej Frant, Katarzyna Podgórska, Anna Gal-Cisoń, Magdalena Łyjak, Kinga Urbaniak, Grzegorz Woźniakowski, Lilla Denes, Gyula Balka, Lapo Nannucci, Amadeu Griol, Sergio Peransi, Zoitsa Basdagianni, Christos Mourouzis, Alessandro Giusti, Ioannis Bossis

**Affiliations:** 1 Laboratory of Animal Husbandry, Department of Animal Production, School of Agriculture, Faculty of Agriculture, Forestry and Natural Environment, Aristotle University of Thessaloniki, 54124 Thessaloniki, Greece; gmanes@agro.auth.gr (G.M.); basdagianni@agro.auth.gr (Z.B.); 2 Department of Swine Diseases, National Veterinary Research Institute, Partyzantów Avenue 57, 24-100 Puławy, Poland; maciej.frant@piwet.pulawy.pl (M.F.); kp@piwet.pulawy.pl (K.P.); anna.gal@piwet.pulawy.pl (A.G.-C.); magdalena.lyjak@piwet.pulawy.pl (M.Ł.); kinga.urbaniak@piwet.pulawy.pl (K.U.); 3 Department of Infectious, Invasive Diseases and Veterinary Administration, Faculty of Biological and Veterinary Sciences, Nicolas Copernicus University in Torun, Lwowska 1, 87-100 Torun, Poland; grzegorz.wozniakowski@umk.pl; 4 Department of Pathology, University of Veterinary Medicine Budapest, Istvan Str. 2, 1078 Budapest, Hungary; denes.lilla@univet.hu (L.D.); balka.gyula@univet.hu (G.B.); 5 National Laboratory of Infectious Animal Diseases, Antimicrobial Resistance, Veterinary Public Health and Food Chain Safety, University of Veterinary Medicine, István Str 2., 1078 Budapest, Hungary; 6 Dipartimento di Scienze e Tecnologie Agrarie Alimentari Ambientali e Forestali, Università Degli Studi di Firenze, Piazzale delle Cascine 18, 50144 Florence, Italy; lapo.nannucci@unifi.it; 7 Nanophotonics Technology Center, Universitat Politècnica de València, Camino de Vera s/n Building 8F, 46022 Valencia, Spain; agriol@upvnet.upv.es; 8 DAS Photonics SL, Camino de Vera, s/n, Building 8F 2nd-Floor, 46022 Valencia, Spain; speransi@dasphotonics.com; 9 Cyprus Research and Innovation Centre Ltd. (CyRIC), 28th Octovriou Ave 72, Off. 301, Engomi, 2414 Nicosia, Cyprus; c.mourouzis@cyric.eu (C.M.); alessandro@cyric.eu (A.G.)

**Keywords:** point of care, diagnostics, photonic integrated circuits, microfluidics, African swine fever virus, classical swine fever virus, oral fluids, validation, sensitivity, specificity, diagnostic odds ratio

## Abstract

Swine viral diseases have the capacity to cause significant losses and affect the sector’s sustainability, a situation further exacerbated by the lack of antiviral drugs and the limited availability of effective vaccines. In this context, a novel point-of-care (POC) diagnostic device incorporating photonic integrated circuits (PICs), microfluidics and information, and communication technology into a single platform was developed for the field diagnosis of African swine fever (ASF) and classical swine fever (CSF). The device targets viral particles and has been validated using oral fluid and serum samples. Sensitivity, specificity, accuracy, precision, positive likelihood ratio (PLR), negative likelihood ratio (NLR), and diagnostic odds ratio (DOR) were calculated to assess the performance of the device, and PCR was the reference method employed. Its sensitivities were 80.97% and 79%, specificities were 88.46% and 79.07%, and DOR values were 32.25 and 14.21 for ASF and CSF, respectively. The proposed POC device and PIC sensors can be employed for the pen-side detection of ASF and CSF, thus introducing novel technological advancements in the field of animal diagnostics. The need for proper validation studies of POC devices is highlighted to optimize animal biosecurity.

## 1. Introduction

By 2050, food demand is expected to increase anywhere between 30% and 62% when considering the climate change scenario [1]. It is also recognized that animal production plays a significant role in food security by providing products of high nutritional value and sustenance for millions of people around the world [2]. More specifically, the swine sector largely contributes to food production, producing around 35% of meat globally [3]. Considering the above, maintaining the supply of protein sourced from animals in a sustainable and efficient manner to meet the increasing demand and safeguard balanced diets can be a challenging task [4,5]. To address this issue, modern animal production focuses on intensification, increased stocking density, and extensive supply chains [2] However, high stocking density and globalized trade networks are known to facilitate pathogen transmission, whereas the scarce surveillance programs often prove insufficient to control animal diseases [6,7].

Among the various pathogens causing diseases in animals, viruses are often difficult to manage due to their transmission dynamics, the non-existent or at best limited treatment options, the lack of vaccines or their efficiency, and the available preventive measures, which are mostly based on hygiene and biosecurity [8,9,10,11]. Such examples in the swine sector are African swine fever virus (ASFV) and classical swine fever virus (CSFV). Both of these viruses can cause disease outbreaks characterized by high mortality and morbidity rates as well as further economic losses, even to unaffected farms, due to restrictions imposed on the trade of live animals and their products.

African swine fever virus (ASFV) is an enveloped, icosahedral DNA virus belonging to the Asfarviridae family. Its genome comprises linear double-stranded DNA, ranging from 170 to 193 kb, with 151–167 closely arranged open reading frames (ORFs) encoded on both DNA strands [12]. The ASFV particle has a multi-layered structure, with an outermost layer resembling an external envelope membrane, although it is not necessary for virus infectivity. Beneath this layer, the capsid is formed by 2760 hexameric and 12 pentameric capsomers, reaching a maximum diameter of 250 nm. Notably, the icosahedral capsid, synthesized by 8280 copies of protein p72 and minor capsid proteins, is of significant interest. P72 constitutes approximately 32% of the viral particle’s total weight and is the primary antigen detected in naturally infected pigs, making it suitable for highly sensitive diagnostic assays [13].

ASFV infection typically manifests as acute hemorrhagic fever in naive populations or as chronic disease in endemic regions. Common symptoms include fever, abortion, skin hyperemia, and hemorrhages in internal organs [14]. Various factors, such as immune system status, infection route, virulence, and virus dosage, influence the disease’s clinical presentation and progression. ASFV can be detected in blood and oropharyngeal samples regardless of virulence; however, oral excretion is sporadic in animals with chronic infections [14,15]. The virus persists in both animal tissues and the environment, facilitating transmission through swill feeding and fomites [16]. Wild boars, susceptible to the virus with symptoms akin to domestic pigs, are considered a potential route for disease transmission. Virus isolation, fluorescent antibody test antigen detection, ELISA, and PCR assays are commonly used to detect ASFV, with PCR being viewed as the standard method due to its superb performance [15]. Currently, there are no commercially available antiviral drugs or vaccines available against ASFV. However, a recombinant experimental vaccine candidate, ASFV-G-ΔI177L, has been developed by deleting the I177L gene from the genome of the highly virulent pandemic ASFV strain Georgia and is currently under trial [17].

Classical swine fever virus (CSFV) is a small RNA virus with an envelope, exhibiting an icosahedral structure and a diameter ranging from 40 to 60 nm. It belongs to the Pestivirus genus within the Flaviridae family. The single-stranded RNA (ssRNA) genome is approximately 12.3 kb long, featuring a solitary open reading frame (ORF) flanked by two untranslated regions (UTRs) [18]. The ORF encodes a sizable polyprotein, which undergoes cleavage by viral and cellular proteases, resulting in the generation of four structural proteins—capsid protein C and envelope glycoproteins Erns, E1, and E2—and eight nonstructural proteins (Npro, p7, NS2, NS3, NS4A, NS4B, NS5A, and NS5B) [19]. The non-structural proteins play a crucial role in cytoplasmic viral replication, with NS5B serving as an RNA-dependent RNA polymerase and NS3 functioning as a protease. Among the structural proteins, E2 is immunodominant, and pigs that recover from CSFV infection produce lifelong neutralizing anti-E2 antibodies [20].

CSFV has the potential to spread through the oronasal route, either directly or indirectly via contact with infected pigs and contaminated feed. Vertical transmission to piglets, either through the placenta or via direct contact with infected sows, is another mode of transmission. Additionally, the virus can be transmitted through insemination and cooled and frozen pork products [21]. CSFV infection typically manifests in three distinct forms: acute, chronic, and persistent. The acute phase, occurring within two weeks since the infection, is characterized by atypical clinical signs initially. Between two to four weeks post infection, neurological signs emerge, including incoordination, paresis, paralysis, and convulsions. Simultaneously, typical symptoms like skin hemorrhages or cyanosis appear on the ears, limbs, and ventral abdomen, often leading to mortality rates as high as 100% [22]. The chronic manifestation of CSFV involves non-specific clinical signs such as remittent fever, depression, wasting, and diffuse dermatitis [22]. The persistent form of the disease is usually associated with mild clinical signs in pregnant sows. Infection between the 50th and 70th day of pregnancy may result in the birth of persistently infected piglets [20]. The diagnostic assays used to detect CSFV are similar to those used for the detection of ASFV.

In many countries, there is a legal framework for the control and surveillance of ASF and CSF, with the diseases being notifiable to the World Organization for Animal Health (WOAH). Disease control strategies involve reliable diagnosis, stamping out infected herds, establishing restriction zones, implementing movement restrictions, and tracing possible contacts [11,20]. For CSF, the decision to implement prophylactic vaccination depends on each country’s policy and relevant epidemiological data [20].

In light of the aforementioned information, it becomes clear that achieving a reliable and early diagnosis is crucial for effective disease control. Presently, the diagnosis or laboratory confirmation of viral diseases relies on methods such as PCR, immunoassays, and, to a lesser extent, cell cultures. However, these techniques demand specialized equipment (e.g., thermocyclers), trained personnel, and frequently centralized laboratories. Consequently, the time from disease onset to laboratory confirmation is prolonged, ranging from days to even weeks.

Specifically for CSF, this period, also known as the high-risk period, is considered a significant risk for facilitating unnoticed spread of the disease. The high-risk period is estimated to last from 2 to 9 weeks, mostly due to great variability in clinical symptoms and the often atypical course of the disease [23]. This often results in diagnoses that do not align with the progression of the disease [24].

To tackle this issue, point-of-care (POC) diagnostic devices have been proposed as effective tools for timely diagnosis, enhancing livestock biosecurity, and addressing animal diseases. POC diagnostics are analytical devices and tests capable of providing rapid on-site diagnosis without reliance on central laboratories [25]. The World Health Organization (WHO) suggests that ideal POC applications should adhere to the “ASSURED” criteria (Affordable, Sensitive, Specific, User-friendly, Rapid and robust, Equipment-free, Deliverable to those who need them) [26]. Common POC tests include dipstick and strip tests, as well as lateral flow assays. However, these tests may face challenges like insufficient sensitivity and relatively high detection limits [27]. Technological advancements, including micro- and nano-fabrication, information and communication technologies, photonics, microfluidics, and advanced materials, have been employed in POC devices and tests to enhance performance and broaden the range of targeted analytes. This has led to the introduction of lab-on-a-chip (LOC) devices, which translate conventional diagnostic techniques such as PCR, LAMP, and ELISA to POC devices [28,29,30]. Despite these advancements, many POC diagnostics for animal diseases encounter limitations, including cost-effectiveness, complexity, extended analytical time, a limited number of targeted analytes, a lack of field testing, and inadequate validation, resulting in the introduction of low-quality tests onto the market [31].

In this context, aPOC device incorporating microfluidics, photonics, and communication technologies was created under the European Union’s H2020 SWINOSTICS (Swine Diseases Field Diagnostics Toolbox) project (https://cordis.europa.eu/project/id/771649, accessed on 1 March 2024). The device is designed for detecting six major swine viral pathogens: porcine reproductive and respiratory syndrome virus (PRRSV), swine influenza A virus (SIV), porcine parvovirus (PPV), porcine circovirus 2 (PCV-2), classical swine fever virus (CSFV), and African swine fever virus (ASFV). The detection of viral antigens is facilitated by photonic integrated circuits (PICs) functionalized with polyclonal or monoclonal antibodies as molecular recognition elements (MREs).

The objective of this study was to present the initial validation results of the innovativePOC device for the detection of ASFV andCSFV in oral fluid and serum samples. Additionally, the study aimed to evaluate the device’s performance by determining crucial performance metrics, including the limit of detection (LOD), sensitivity, specificity, accuracy, precision, positive likelihood ratio (PLR), negative likelihood ratio (NLR), and diagnostic odds ratio (DOR).

## 2. Materials and Methods

### 2.1. Samples

The ASFV strain (Pol18_28298; virus titre 10^6^ HAD_50_/mL), which was shared by the National Veterinary Research Institute (NVRI/PIWet) in Pulawy (Poland) for the purposes of this study, was isolated from the ASF domestic pig outbreak in Poland no. 111 (date of confirmation: 22 May 2018; sample location: voivodship—Lubelskie, poviat—Chełm, municipality—Sawin). The reference strain Pol18_28298 belongs to genotype II. The primary material was collected by local employees of the Veterinary Inspection within the frame of the ASFV monitoring program in Poland. For virus isolation and propagation, a method previously described was applied [32]. Similarly, virus titration and quantification were determined as previously described [32]. The virus strain was heat-inactivated at 56 °C for 70 min. The CSFV strains used in the study included reference strain Alfort 187, as well as historical field strains from Poland, Estonia, and Germany, which were used for tests conducted in Poland. All strains were propagated in the SK-6 cell line based on the standard procedure as previously described in the WOAH Manual of Diagnostic Tests and Vaccines for Terrestrial Animals, chapter 3.9.3. Classical swine fever, 12th edition 2023.

Oral fluid samples were retrieved from four countries: Poland, Greece, Italy, and Hungary. Serum samples were collected as part of standard health monitoring practices implemented on commercial swine farms. All samples were transported to the laboratory at 4–6 °C and processed within 24 h. Oral fluids were freeze–thawed and centrifuged at 12,000× *g* for 10 min, and supernatants were stored at −80 °C. Serum samples were centrifuged at 800–1500× *g* for 5–10 min and supernatants were also stored at −80 °C. ASF-functionalized sensors were tested with 9 negative and 18 positive (11 spiked and 7 clinical) oral fluid samples. Additionally, ASF-functionalized sensors were tested with 36 positive serum samples (22 spiked and 14 clinical) for the assessment of the performance of the novel POC device. To estimate the limit of detection (LOD) of the ASF-functionalized sensors, six serial 3-fold dilutions of the reference ASF samples (Pol18_28298) in serum and oral fluids were used. CSF-functionalized sensors were tested with 47 negative and 33 positive (31 spiked and 2 clinical) oral fluid samples. Six serial 3-fold dilutions of the CSF reference sample in oral fluids were used for the estimation of the LOD of CSF-functionalized sensors. All clinical samples were tested in Poland. The status (negative or positive) of all samples underwent laboratory confirmation using quantitative (reverse transcription for CSF) PCR assays.

### 2.2. DNA/RNA Extraction and PCR Assays

Viral DNA from ASF and RNA from CSF were extracted using the PureLink™ Viral RNA/DNA Mini Kit (Invitrogen, Carlsbad, CA, USA). The DNA/RNA isolation protocol adhered to the manufacturer’s guidelines, with a standard volume of 200 μL per sample. The elution of nucleic acids from each sample was carried out in 20 μL of elution buffer and subsequently stored at −80 °C. Reverse transcription of total CSF RNA into cDNA was conducted using random primers and the High-Capacity cDNA Reverse Transcription Kit with RNase Inhibitor (Applied Biosystems™, Vilnius, Lithuania) with standard reaction volumes of 20 μL (10 μL sample and 10 μL kit reagents), following the manufacturer’s instructions. The resulting cDNA was stored at −80 °C.

The detection of ASF virus DNA was performed by conventional PCR with a primer set (ASF_Set_1) targeting the VP72 gene. For CSF virus RNA detection, nested conventional PCR was utilized with four primer sets targeting the E2 gene and 5′ NTR. All conventional PCR assays were conducted in a total volume of 25 μL, comprising 22.5 μL of PCR 1.1 × SuperMix (Invitrogen, Carlsbad, CA, USA), 0.5 μL of 10 μM forward primer solution, 0.5 μL of 10 μM reverse primer solution, and 1.5 μL of template DNA or cDNA. The cycling conditions included pre-denaturation at 95 °C for 2 min, followed by 32 cycles of denaturation at 94 °C for 20 s, annealing for 30 s, extension at 72 °C, and a final extension at 72 °C for 1 min. Optimized annealing temperatures and extension times for each primer set are provided in Table 1. Subsequently, PCR products were analyzed in a 2% agarose gel and stained with ethidium bromide. A 100 bp ladder (Thermo Scientific, Vilnius, Lithuania) was utilized to determine amplicon length.

Quantification of viral DNA and cDNA through real-time PCR was carried out in triplicate using SYBR Green chemistry. For ASF and CSF, the primer sets utilized were ASF_Set_1 (targeting the VP72 gene) and CSF_Set_1_Nested_2 (targeting the E2 gene), respectively. The reactions were conducted in a total volume of 20 μL, comprising 10 μL 2 × PowerUp™ SYBR™ Green Master Mix with 500 nm ROX (Applied Biosystems, Vilnius, Lithuania), 0.5 μL of 10 μM forward primer solution, 0.5 μL of 10 μM reverse primer solution, 1 μL of template viral DNA or cDNA, and 8 μL H_2_O. The cycling conditions included an initial activation of UDG for 2 min at 50 °C, activation of the Dual-Lock polymerase for 2 min at 95 °C, followed by 40 cycles of denaturation at 95 °C for 15 s and annealing and extension at 60 °C for 1 min. Data collection was performed using a 7500 Real Time PCR System, and analysis was carried out with 7500 software, v.2.0.6 (Applied Biosystems).

Viral load quantification involved the use of standard curves derived from known DNA amounts, ranging from 10^10^ for CSFV and 10^9^ for ASFV down to 10^3^ viral genome copies per PCR reaction, all in duplicate. The DNA utilized to construct the standard curves was derived from purified PCR products following gel electrophoresis. For the determination of DNA content, all samples underwent quantification through photometry (Quawell Q5000, San Jose, CA, USA). The copy number was calculated based on the DNA content, the average molecular weight of deoxyribonucleotides, the number of deoxyribonucleotide bases for each DNA product, and Avogadro’s number. Viral concentrations were expressed as the viral copy number per ml of the sample.

### 2.3. POC Device, Antibodies, and Sensors

**Figure 1:**
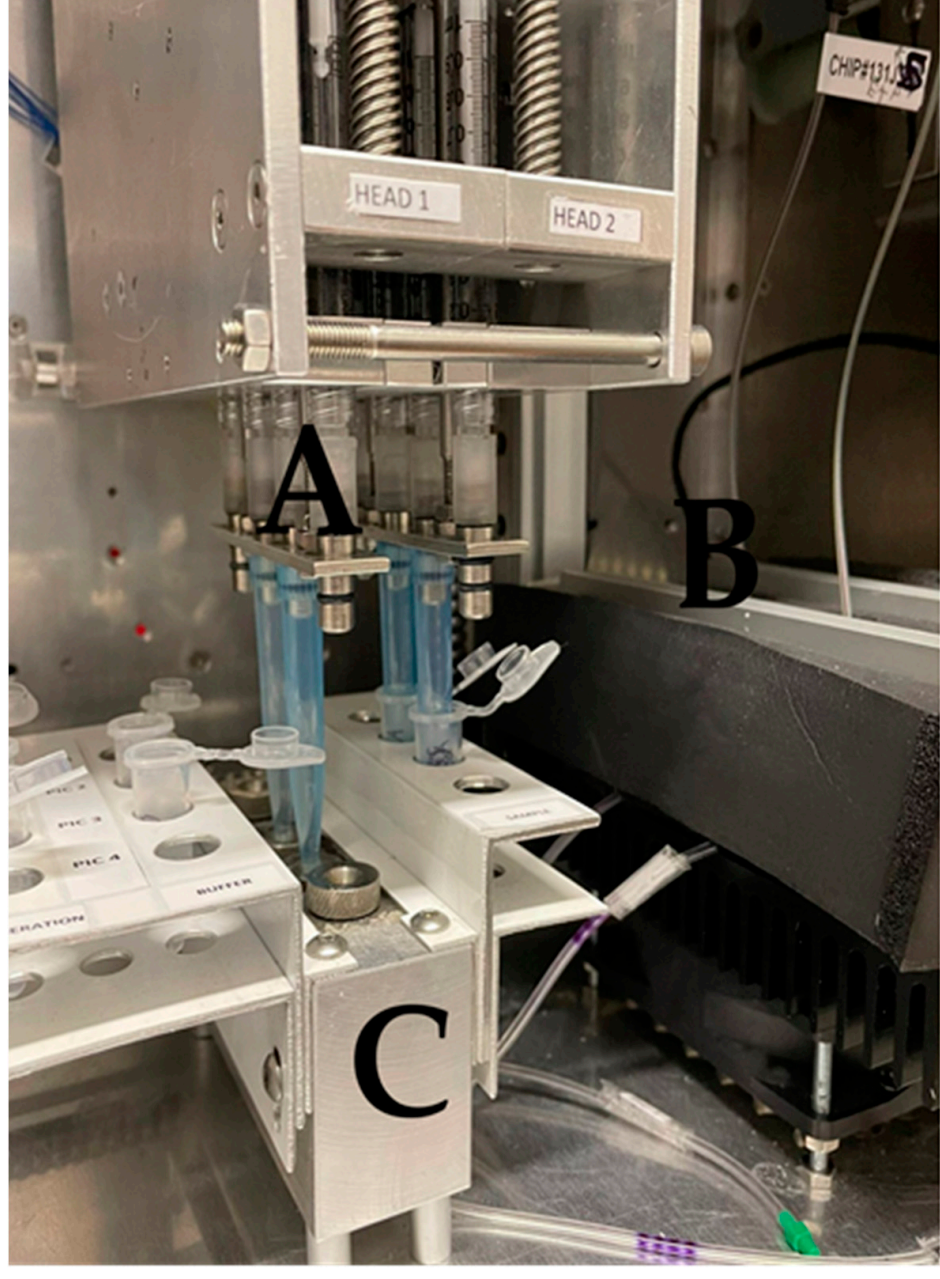
The novel POC device. (**A**) Syringe system for buffer/sample delivery. (**B**) Insulating material that covers the PICs and in combination with the Peltier element maintains a steady temperature of 25 °C during analysis. (**C**) Sample and buffer holder. The circular metallic inlets of the microfluidic system are shown.

A detailed description of the POC device has been previously published [2,35]. The novel POC device utilizes microfluidics and PICs for the detection of ASF and CSF. A moveable arm, equipped with syringes, was used to deliver buffers and samples to PICs through microfluidic channels as a cost-effective alternative to peristaltic pumps (Figure 1). A Peltier element was used to control the temperature during the analysis. PICs were also coupled with a tunable laser and a photodiode for optical signal detection. Paired data of the laser’s wavelength and photodiode responses were recorded using an Arduino data logger. A microcontroller was used to operate the device and establish Bluetooth communications with a tablet/smartphone through an Android application. The recorded data were delivered to a cloud platform that can generate simple yes/no results in real time. The assay could be completed within 60 min. Minimal training and handling were required for device operation, and end-users only had to add pipette tips and buffers/samples to the device. The device was able to analyze up to 4 samples simultaneously (each sample can be tested for 2 of the 6 diseases in one run), enabling multiplexing. Finally, all modules were integrated in a single portable device weighing 45 kg and with dimensions of 40 × 50 × 60 cm. To date, the device has undergone initial field testing in commercial swine farms in Greece, Italy, and Hungary.

**Figure 2:**
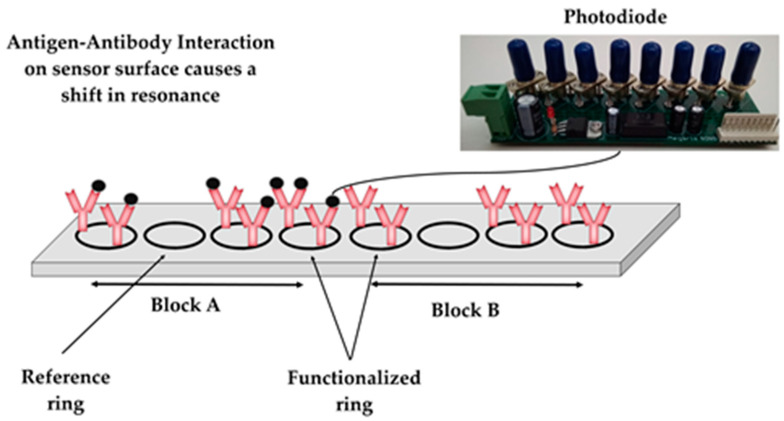
Sensor concept and setup.

The PIC sensors and their validation process have been previously described [36,37]. Each PIC sensor utilizes 2 blocks of ring resonators. Each block consisted of 4 ring resonators that detect a given swine pathogen (Figure 2). Three out of the four ring resonators were functionalized with antibodies for the capture of the targeted virus particle. The fourth ring’s surface was functionalized with fish gelatin and served as a reference. Antigen–antibody interactions (bonding) caused a localized change in the refractive index extending beyond the sensor’s surface [38]. Consequently, the additional mass (captured antigen) shifted the resonant wavelength of antibody-functionalized rings. For the detection of ASFV in the samples, a monoclonal anti-VP72 protein antibody (M.11.PPA.I1BC11, Ingenasa, Madrid, Spain) was selected for the functionalization of the sensors. Correspondingly the detection of CSFV in the samples, a polyclonal anti-E2 envelope protein antibody (CSFE21-S, Alpha Diagnostic, San Antonio, TX, USA) was used.

### 2.4. Analysis Protocol and Shift Calculation

The analysis protocol was optimized for the detection of ASFV and CSFV in complex biological matrices (oral fluids and sera). A detailed description of the analysis protocol has been published previously [2,35]. During the analysis, buffers and samples were propelled to the sensors at a steady flow rate of 30 μL/min. Outflows were delivered to a waste tank for UV sanitization. The protocol is presented in detail in Table 2.

**Figure 3:**
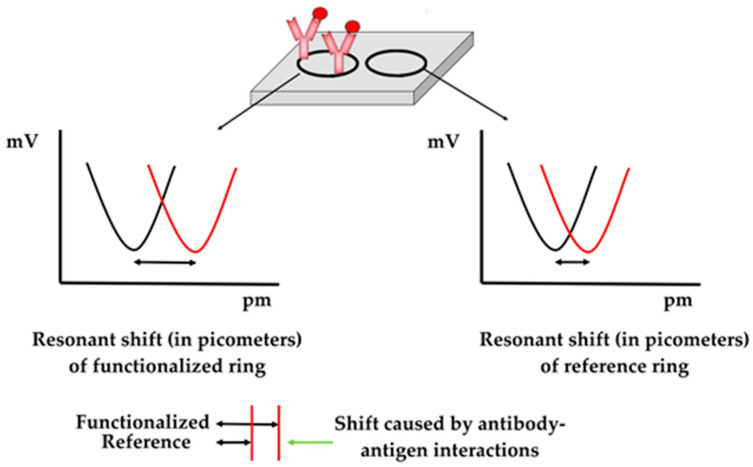
Shift calculation principle. The black lines in the plot represent the state of the sensor (minimum) prior the introduction of the sample, whereas the red lines represent the state of the senor (minimum) after introducing the samples.

Resonant shifts were calculated using an algorithm, accessible through an Android application a cloud platform. The LOWESS algorithm [39] was utilized to smoothen the scatterplot of mV values versus the laser wavelength values and facilitate shift calculation for each ring independently. The resonant shift in pm caused by the antibody–antigen interactions was calculated by subtracting the resonant shift of reference rings (absolute values in pm) from the resonant shift of antibody-functionalized rings (absolute values in pm) (Figure 3). Shift values greater than 0 are considered as positive results (detection of viral particles), whereas values of 0 or lower are considered as negative results (absence of viral particles).

### 2.5. Limit of Detection (LOD) Experiments

ASFV and CSFV reference samples were quantified using the previously described qPCR assays. To estimate the LOD of ASF-functionalized sensors, six serial 3-fold dilutions (range of 10^7^–3.3 × 10^4^ copies/mL) of the reference sample diluted in oral fluids were tested with 2 sensors. Additionally, the LOD of ASF-functionalized sensors in sera was tested with 4 sensors, by using six serial 3-fold dilutions (range of 10^7^–3.3 × 10^4^ copies/mL). To estimate the LOD of CSFV-functionalized sensors, six serial 3-fold dilutions (range of 10^8^–3.3 × 10^5^ copies/mL) of the reference sample added to oral fluids were tested with 7 sensors.

### 2.6. Validation and System Performance

The LOD experiments (presented in detail in Section 3) revealed that shift values could not be fit to a linear model with respect to the viral copies per mL. Consequently, a qualitative system with a binary response variable (positive or negative) was adopted. Initially, receiver operating characteristic (ROC) curves were drawn to calculate the optimal threshold (shift value in pm that achieves the best combination of sensitivity and specificity), the area under the curve (AUC), and the respective 95% confidence intervals (95% CI) for both ASFV and CSFV, using SPSS v23 software (IBM Corp., Armonk, NY, USA). Test outcomes, i.e., true positives (TP), true negatives (TN), false positives (FP), and false negatives (FN), were calculated using the optimal threshold of the ROC analysis and calibrators (samples) representing three categories, as previously suggested [40]. Negative (N) calibrators were considered the samples that tested negative with conventional and real-time PCR methods. Low positive (LP) calibrators were considered the samples with Ct values equal to or higher than 30. Positive (P) calibrators were considered samples that had Ct values lower than 30 in real-time PCR.

In the second stage, the test outcomes were used to evaluate the diagnostic performance of the device by estimating its sensitivity, specificity, accuracy, precision, positive likelihood ratio, and negative likelihood ratio, as well as their 95% confidence intervals (95% CI). The calculations were performed using the MedCalc online software (https://www.medcalc.org/calc/diagnostic_test.php, accessed on 4 September 2022). To provide a prevalence-independent global estimator of the discriminative power of the device that allows direct comparisons between different diagnostics tests, the diagnostic odds ratio (DOR = ((TP/FP)/(FN/TN))) was calculated [41]. The diagnostic odds ratio of a test is the ratio of the odds of positivity in the positive group, relative to the odds of positivity in the negative group. The 95% CI of the DOR was calculated using the formula Log(DOR) ± 1.96SE(Log(DOR)), where SE(Log(DOR)) = √(1/TP + 1/TN + 1/FP + 1/FN) [41].

The ASFV samples were tested with a total of 17 PICs and the CSFV samples were tested with 20 PICs. PICs were used up to six times and additional experiments were not attempted due to the structural deterioration of the sensors after excessive use. ASF-functionalized PICs provided 177 valid results at the ring level, whereas CSFV PICs provided 272 valid results. Considering that each ring resonator functions independently, the validation of the POC device was conducted at the ring level.

## 3. Results

### 3.1. PCR Results

**Figure 4:**
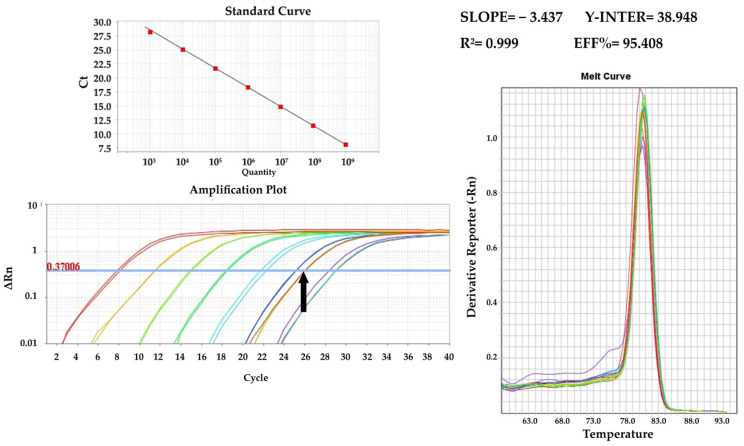
qPCR standard curve using the ASF_Set_1 (VP72 gene) primer set and the amplification plot with reference samples indicated with the black arrow.

**Figure 5:**
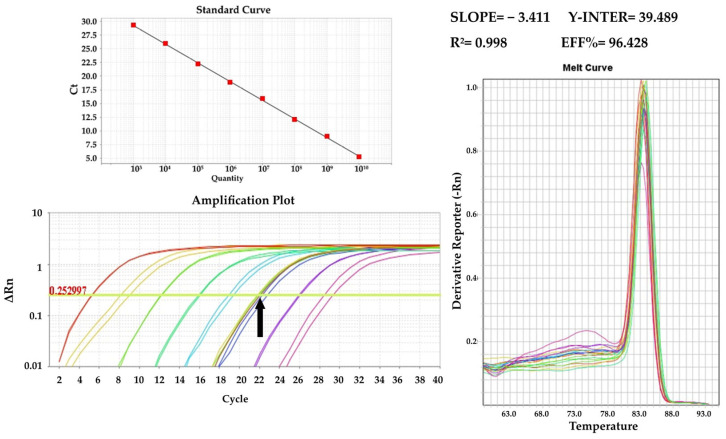
qPCR standard curve using the CSF_Set_1_Nested2 (E2 gene) primer set and the amplification plot with reference samples indicated with the black arrow.

The screening of samples involved both the conventional and real-time PCR assays mentioned earlier. All samples included in this study adhered to the following qualification criteria: (i) negative samples for a specific disease should yield negative results with all available primers for that disease and (ii) positive samples should test positive with all available primer sets for the respective disease. Reference samples, along with those utilized in the limit of detection (LOD) experiments, underwent quantification using SYBR Green real-time PCR assays and the standard curves provided below (Figure 4 and Figure 5).

### 3.2. Limit of Detection (LOD)

**Figure 6:**
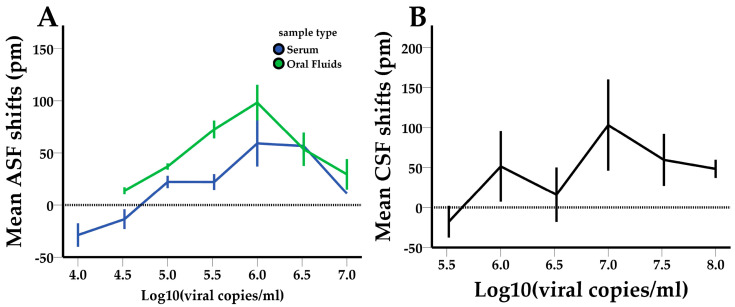
(**A**) ASFV and (**B**) CSFV shift responses (in pm) plotted against viral concentrations (Log_10_(viral copies/mL)). The results were obtained from the LOD experiments.

In the image below (Figure 6), the shift responses in pm of the ASF- and CSF-functionalized PICs are plotted against the corresponding viral concentrations (in Log_10_(viral copies/mL)) of the samples. The error bars represent the standard errors of the shifts in each viral concentration. The lowest detectable viral concentration (LOD) is indicated by shift values that approach zero. ASF- and CSF-functionalized sensors showed an LOD of approximately 3.3 × 10^4^ viral copies/mL and 3.3 × 10^5^ viral copies/mL, respectively. For both viruses, the prozone effect prevents dose-dependent shift responses, thus leading to the adoption of a qualitative response (yes or no) system. Additionally, ASFV-spiked oral fluids (green line) produce greater shift responses than ASFV-spiked sera (blue line), indicating that oral fluids are more suitable for photonic measurements and the current system setup. Finally, the lower LOD values of ASF-functionalized sensors may be correlated with their production at a later stage and the exploitation of previous experience in their manufacture.

### 3.3. Receiver Operating Characteristic (ROC) Curve

**Figure 7:**
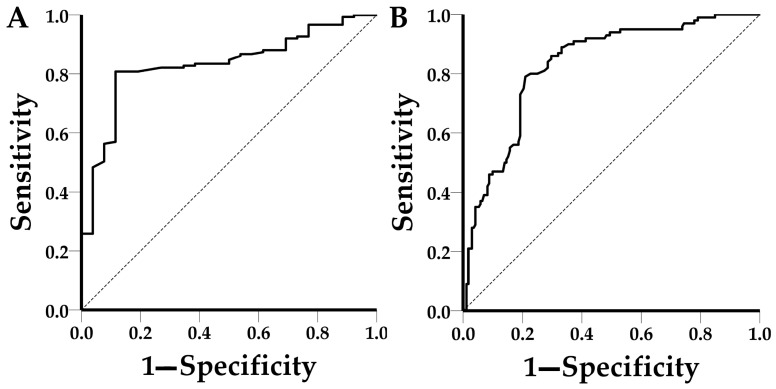
ROC curves of (**A**) ASFV, AUC: 0.832 (95% CI: 0.758–0.906) and (**B**) CSFV, AUC: 0.830 (95% CI: 0.781–0.880).

For the estimation of the area under the curve (AUC) values and the optimal detection threshold, the 177 and 272 valid results at the ring level were used for ASFV and CSFV, respectively. Youden’s index (=sensitivity + specificity − 1) was used to identify the optimal detection threshold. ASFV-functionalized sensors achieved an AUC value of 0.832 (95% CI: 0.758–0.906) and an optimal shift detection threshold of 5.2 pm, which corresponds to 80.8% sensitivity and to 88.5% specificity (Figure 7). Correspondingly, the CSFV-functionalized sensors achieved an AUC value of 0.830 (95% CI: 0.781–0.880) and an optimal shift detection threshold of 5.5 pm, which corresponds to 79% sensitivity and 79.1% specificity (Figure 7).

### 3.4. Validation and System Performance Results

The test outcomes (TP, TN, FP, and FN) obtained by the novel POC device are summarized in Table 3. The performance metrics (sensitivity, specificity, accuracy, precision, PLR, NLR, and DOR), along with their 95% CI, are shown in Table 4.

## 4. Discussion

This study showcases the integration of photonic integrated circuits (PICs), microfluidics, and communication technologies into a single device designed for the detection of swine viral diseases in oral fluids and serum samples. While the sensitivity and selectivity of PICs have been previously harnessed in applications such as gas sensing, biomedical diagnostics, and biochemical detection [42,43,44], this marks the first effort to leverage PICs for detecting swine viral pathogens in a POC setting. The novel device achieved LOD values of 3.3 × 10^4^ viral genome copies/mL for ASF and 3.3 × 10^5^ viral genome copies/mL for CSF. In general, PIC performance was satisfactory at the ring level. Sensitivity was around 80%, but specificity was somewhat higher in ASF (88.46%) than in CSF (79.07%). Accuracy was similar between the two types of sensors, but precision was statistically higher in ASF sensors. PLR was higher in ASF sensors (not a statistically significant difference) and the NLR was similar between the two types of sensors. Finally, the DOR values were higher in the ASF sensors (DOR_ASF_ = 32.25 vs. DOR_CSF_ = 14.21).

For comparison, in a separate investigation, an integrated microfluidic platform designed for the multiplex detection of circulatory antibodies against PRRSV, CSFV, and PCV-2 in serum demonstrated sensitivity values of 89.74%, 96.61%, and 88.89%, respectively. The platform also exhibited specificities of 96.61%, 97.22%, and 98.31%, accuracy values of 93.88%, 96.84%, and 94.74%, and AUC values of 0.968, 0.992, and 0.989 when evaluated with a sample size of 100 [30]. However, direct comparisons with the present study are challenging due to the absence of 95% confidence intervals for establishing statistically significant differences. Additionally, the referenced study lacks adequate information about the sample characteristics and whether they encompassed a broad range of analyte concentrations.

Increased research interest in POC applications for detecting ASFV has surged due to its significant impact on the global swine sector. Various attempts have been made to develop POC ASF detection methods, broadly categorized into protein-based and nucleic acid-based approaches. One such attempt resulted in the development of a rapid lateral flow assay with 99.8% specificity and 84.52% sensitivity for ASF detection [45]. Another study presented a lateral flow assay with 59.1% sensitivity (95% CI, 49.1–69.1) and 91.7% specificity (95% CI, 84.7–98.7) when tested with tissue samples [46]. In the nucleic acid-based category, a study evaluated the TripleE system for nucleic acid extraction in field conditions followed by PCR testing using thermal cyclers suitable for field operations [47]. However, this approach yielded moderate results, particularly in samples with Ct values over 30. Additionally, loop-mediated isothermal amplification (LAMP) assays have been proposed for ASF testing in field conditions. One LAMP-based POC application demonstrated 100% sensitivity and specificity [48], though details about the samples were lacking. Another study assessed a microfluidic LAMP chip for ASF detection, achieving 100% sensitivity and a limit of detection (LOD) of approximately 10^5^ copies/mL [49], albeit solely tested with purified nucleic acids and negative serum samples. Further advancements include recombinase-aided amplification combined with a lateral flow assay, achieving LOD values of 10^4^ copies/mL [50], although the approach required nucleic acid isolation before testing. A well-designed study utilizing a portable microfluidic-circular fluorescent probe-mediated isothermal nucleic acid amplification (CFPA) system showed sensitivity of 92.73% and 100% specificity for ASF detection [51]. Additionally, a Cas12a-based assay coupled with recombinase polymerase amplification and a fluorophore-quencher labeled reporter assay has been proposed for ASF detection [52]. However, the approach was tested with purified nucleic acids and necessitated specialized equipment. In summary, while lateral flow assays offer rapid and cost-effective detection with minimal equipment and personnel requirements, they often lack sensitivity, especially in the early stages of infection. Conversely, nucleic acid-based methods provide high sensitivity and specificity but require multiple steps, nucleic acid isolation, and specialized training and equipment.

Recognizing the pivotal role of reliable POC diagnostics in controlling swine viral diseases and mitigating their socioeconomic impact, the novel device addresses a significant limitation of these tests—the potential decrease in performance by untrained individuals. To overcome this challenge, the device was designed to fully automate the entire analysis process, including fluid delivery, measurements, and data analysis for disease detection. The operational steps involve oral fluid collection using cotton ropes, sample dilution, and filtration. Users are then required only to place sensors and add pipette tips, buffers, and samples, thus minimizing user-introduced bias in photonic measurements. Device operation is facilitated through a tablet employing a user-friendly Android application. Automation extends to data analysis, providing straightforward positive or negative results. As a result, the device can be effectively utilized by non-specialized personnel, with minimal impact from mishandling.

Results from the device were stored online via a cloud platform and suitable data transfer applications, enabling meta-analysis and the establishment of effective surveillance protocols for targeted diseases. These technological advancements hold promise for the evolution of telemedicine in animal production. The system’s modular approach facilitates the easy servicing and replacement of components. The overall system architecture allows for potential deployment on farms, peripheral and mobile laboratories, as well as border checkpoints.

The efficacy of the bio-recognition event on the sensor’s surface is influenced by the selection of appropriate antibodies. Antibodies were meticulously chosen to target conserved viral proteins, ensuring detection capability across a broad spectrum of circulating viral strains. Buffer solutions incorporated mild detergents to partially disassemble the virus envelope, facilitating the recognition of antigens on the virions. Although the antibodies in this study were not oriented for functionalization, specifically binding the antibody Fc region to the sensor surface, the quantity of antibodies used proved sufficient for the successful occurrence of the bio-recognition event.

Throughout this study, device validation and performance metric calculations were conducted at the ring level. For each PIC, three ring resonators were functionalized for a specific pathogen. Aggregating information from all three rings for each targeted analyte significantly improved overall performance, yielding approximately 90% sensitivity and specificity at the test level for all viruses, comparable to results in other studies [30]. This approach considered the response of the majority of functionalized ring resonators as the valid result [2]. For instance, if two out of three functionalized rings for a disease yielded negative outputs and one provided a positive output, the valid test result would be considered negative (indicating the absence of the targeted analyte and no detection). While this approach is effective, it deviates from the initial plan of using a single ring resonator for each disease, which would enable the multiplex detection of all six targeted pathogens with a single sensor.

The limit of detection (LOD) values achieved in this study were adequate for identifying clinical cases of the targeted diseases. Notably, the samples were directly utilized in the device without any pretreatment or sample enrichment steps. In contrast, PCR and RT-LAMP assays transformed into lab-on-chip devices using microfluidic chips exhibited LOD values of 10^3^ or 10^4^ viral genome copies/mL, respectively [29,30]. However, both assays necessitated the labor-intensive laboratory-based isolation of nucleic acids as a sample pretreatment step. As mentioned earlier, the device detects fully or partially assembled particles, implying that using PCR as a reference method likely overestimates the viral load in samples used for LOD experiments. This discrepancy is a recognized challenge in virology, as viral genome copies may not always correspond to the number of infectious virions [53]. It is important to note that the prozone effect prevents dose-dependent shift responses, leading to non-linear shift responses with respect to viral concentrations. The phenomenon was observed in both the ASFV and CSFV curves, and thus, a qualitative (yes/no) system was adopted.

The recorded counts of TP, FN, TN, and FP depend on (i) the chosen threshold classifying the device’s response as positive or negative, (ii) inherent device characteristics (including antibodies, the analysis protocol, mechanics/microfluidics, photonics, algorithms, etc.), and (iii) the balance between tested positive and negative samples (e.g., a limited number of positive samples may result in fewer false negatives). ROC curves and Youden’s index were utilized to determine the signal threshold offering the optimal combination of sensitivity and specificity. The practicality of ROC curves in assessing the sensitivity–specificity tradeoff is unquestionable, aiding in establishing desired levels based on disease characteristics and epidemiology. For instance, a POC test for the ASF virus should prioritize high sensitivity, emphasizing high negative predictive values, given the severe consequences of missing positive cases.

While initial impressions may suggest suboptimal sensitivities and specificities, it is crucial to consider the use of low positive calibrators (samples with low copy numbers and Ct values equal to or exceeding 30) in the study. Including low positive samples helps avoid disease spectrum bias, preventing the overestimation of device performance. Sensitivity and specificity values may vary when the test is conducted in settings or populations different from the validation study. Evaluating the performance of a diagnostic test should consider both sensitivity and specificity simultaneously. Furthermore, these two metrics alone are insufficient for assessing thepost-test probability and interpreting test results [54]. Therefore, it is crucial to calculate additional performance metrics to offer a more comprehensive understanding of the diagnostic test’s performance.

Accuracy, as a performance metric, considers both pre- and post-test probabilities, representing the proportion of true classifications among all recorded classifications. While accuracy provides a single metric for evaluating diagnostic tests, it is somewhat influenced by disease prevalence and treats false positives and false negatives equally. Moreover, accuracy is sensitive to variations in the study population and setting, making comparisons based solely on accuracy potentially misleading. The ASF sensors exhibited statistically significantly higher precision compared to the CSF sensors. However, this discrepancy is likely influenced by the ratio of positive and negative samples in the ASF group. In practice, increased prevalence in the screened population enhances precision by reducing false positives, as fewer negatives are tested. Conversely, low prevalence leads to reduced precision by increasing the number of false positives. In another study, two SARS-CoV-2 nucleic acid amplification tests (PCR-based) used for screening in a low-prevalence (0.14–0.41%) population achieved precision values of 61.8–89.8% and 20.1–73.8% [55]. Both precision and negative predictive values are susceptible to prevalence differences, making them unsuitable for evaluating diagnostic tests in different populations. In many cases, the absence of surveillance epidemiological data for swine diseases across animal groups, farms, regions, and/or countries renders precision and negative predictive value impractical for estimating post-test probability in animal POC diagnostics [2].

To address the challenge of prevalence-induced bias in estimating post-test probabilities through precision and negative predictive value, prevalence-independent markers of diagnostic performance, such as PLR and NLR, have been proposed. PLR and NLR serve the purpose of connecting pre- and post-test probabilities by indicating how many times a specific test result is likely to occur in the “diseased” group compared to the “healthy” group. PLR values greater than one suggest a true association with the presence of disease, while NLR values lower than one indicate an association with the absence of disease [56]. A general guideline is that likelihood ratios above 10 or below 0.1 are considered sufficient to confirm or exclude a disease, respectively [56].

The diagnostic odds ratio (DOR) is not likely to be a constant specific to a particular test; instead, its primary utility lies in serving as a comprehensive measure for the diagnostic performance of a test. It can be applied to compare diagnostic tests across populations, irrespective of disease prevalence, making DOR suitable for meta-analyses [41]. However, like other performance metrics, the DOR is still influenced by the disease spectrum in population studies, including the inclusion of low positives. Furthermore, the DOR is not defined in 2 × 2 tables containing zeros, and two tests with identical DOR values can exhibit significant differences in sensitivity and specificity [41].

To comprehensively evaluate the utility and performance of a diagnostic test, it is essential to calculate all of the mentioned metrics. Additionally, well-designed POC validation studies should outline the decision-making framework and post-test result interpretation. However, this is challenging, as it requires detailed information on targeted diseases, epidemiological data, and surveillance systems, which can be costly and time-consuming to acquire. The animal POC diagnostics market and profit margins in animal production often cannot justify the expense of extensive surveillance programs. Consequently, POC devices and tests validated in laboratory settings may prove ineffective in practice, as the appropriate diagnostic tool may not be used in the right framework or setting. For instance, a test with 95% sensitivity and specificity would yield positive predictive values of only 50% in a population with 5% disease prevalence, rendering the hypothetical test unsuitable for ruling in the disease with a positive result. This has a significant impact, as end-users, such as veterinarians or farmers, rely on positive predictive values for making evidence-based decisions. Therefore, POC manufacturers should not solely aim for “perfect” sensitivity and specificity values but also focus on providing the proper framework to maximize the effectiveness of POC testing.

Additionally, many POC tests for animal (and sometimes human) diseases lack adequate validation through field trials and clinical utility assessments, reducing the financial motivation for commercial exploitation due to the elevated risk of test failure [57]. This results in a cycle of limited investment, subpar tests, and a scarcity of innovative POC devices. The situation is exacerbated in animal production by the narrow profit margins for both farmers and commercial companies introducing POC devices. Since the presented diagnostic device has not undergone thorough field validation, attempts at commercialization have not been pursued.

Considering the aforementioned factors, it is recommended that validation studies for innovative POC devices concentrate on three key aspects: (i) conducting proof-of-concept experiments with reference samples; (ii) extensive laboratory testing using a range of negative and positive samples that encompass the entire spectrum of the disease to calculate device performance metrics; and (iii) field testing to assess the practical utility of the device for stakeholders [2]. It is noteworthy that while the novel POC device has undergone limited field testing, this study primarily focused on establishing the proof-of-concept for the proposed system, along with the initial laboratory experiment using complex sample matrices (oral fluids and serum).

In general, the system has the potential to decrease screening expenses and streamline the process of diagnosing viral diseases. The estimated cost for analyzing a single sample (screening for two diseases) is currently EUR 0.60. The device can concurrently test up to four samples, completing the process in about 1 h. A key advantage of the device is its ability to operate directly on farms, in the real POC setting. This allows for the assessment of the health status of animals just before or during the early stages of the disease, facilitating evidence-based disease control strategies.

The device places a primary emphasis on utilizing oral fluids for detecting the targeted diseases. Oral fluids offer advantages such as being non-intrusive, easy to collect, cost-effective, and suitable for herd screening [58,59,60]. While oral fluids are undeniably valuable, other sample types like serum, fecal samples, or nasal swabs can also be considered with adjustments to the analysis protocol, including sample pre-treatment, different dilution factors, and alternative buffers. Exploring alternative sample types is crucial, as the use of antibodies recognizing various antigens (viruses, circulatory antibodies, etc.) typically present in other sample types could potentially broaden the range of analytes detectable by the novel POC device. Therefore, the investigation of alternative sample types should remain a priority.

Subsequent investigations into the proposed concept should concentrate on four key elements: device size, MREs, PICs, and expanded study size. Efforts are underway to reduce the device’s size and increase portability. The exploration of different antigen epitopes using alternative MREs may enable the identification of additional viral strains, viruses, or other analytes, broadening the spectrum of detectable diseases and enhancing the device’s performance. In this context, alternating the MREs deployed on the PIC surfaces the device has the theoretic capacity to detect other analytes including antibodies (serology). Therefore, a potential application, besides a POC test for field use, could be the use of the device as a diagnostic platform to differentiate naturally infected animals from vaccinated animals (DIVA vaccines) or even be used as a quick screening test for viral antigens that induce an immune response to animals, further contributing to reduced vaccine development times.

Continuous epidemiological surveillance and the utilization of antibodies capable of recognizing the prevalent viral strains are essential for ensuring that the device remains up to date. Furthermore, enhancing the performance and quantification capabilities of the device can be achieved through the oriented immobilization of antibodies. The incorporation of 3D microprinters in the functionalization process, applied to both antibodies (detection ring resonators) and blocking proteins (reference ring resonators), can mitigate background interference and enhance signal resolution. Standardizing materials and procedures and implementing fully automated PIC fabrication processes can further minimize tolerances in PIC manufacturing, consequently improving overall device performance.

## 5. Conclusions

Point-of-care (POC) devices play a crucial role in enhancing livestock biosecurity by offering rapid, cost-effective, and reliable tests for diagnosing animal diseases and identifying risk factors in field conditions. This study integrates photonics, microfluidics, and information and communication technologies into a portable device, representing a significant advancement in animal POC diagnostics. Initial validation indicates the novel device’s promising performance, potentially reducing time and costs associated with diagnosing swine viral diseases and facilitating prompt, locally informed decisions for evidence-based disease control measures.

## Figures and Tables

**Table 1:** Primer sets used in conventional PCR for the detection of ASFV and CSFV and optimized annealing temperatures and extension times.

Primer Set	Target Region	Primer Sequence (5′-3′)	Amplicon Length (bp)	Reference	Annealing for 30 s at	Extension at 72 °C
ASF_Set_1	VP72 gene	Forward: GGTTGGTATTCCTCCCGTG Reverse: GATTGGCACAAGTTCGGAC	326	[33]	58 °C	40 s
CSF_Set_1_Nested1	E2 gene	Forward: AGRCCAGACTGGTGGCCNTAYGA Reverse: TTYACCACTTCTGTTCTCA	671	[34]	52 °C	50 s
CSF_Set_1_Nested2	E2 gene	Forward: TCRWCAACCAAYGAGATAGGG Reverse: CACAGYCCRAAYCCRAAGTCATC	272	[34]	58 °C	40 s
CSF_Set_2_Nested1	5′ NTR region	Forward: CTAGCCATGCCCWYAGTAGG Reverse: CAGCTTCARYGTTGATTGT	421	[34]	52 °C	50 s
CSF_Set_2_Nested2	5′ NTR region	Forward: AGCTCCCTGGGTGGTCTA Reverse: TGTTTGCTTGTGTTGTATA	272	[34]	50 °C	40 s

**Table 2:** Description of the analysis protocol for the detection of ASFV and CSFV. The purpose, timing, and buffers at each step are presented.

Analysis Step	Purpose of Step	Time of Step	Buffer for ASFV Detection	Buffer for CSFV Detection
Buffer step	Photonic signal stabilization and baseline establishment	15 min	PBS + 0.05% *v*/*v* Tween 20 + 1% *w*/*v* BSA, pH = 7.4 (T-PBS/BSA)	MES 0.1 M + 1% *w*/*v* BSA, pH = 6 (MES/BSA)
Sample step	Testing and binding of the targeted analytes on functionalized PIC surfaces	10 min	The sample (300 μL) was diluted at a ratio of 1:1 with T-PBS/BSA	The sample (300 μL) was diluted at a ratio of 1:1 with MES/BSA
Washing step	Removal of unbound viral particles and sample residues	15 min	T-PBS/BSA	MES/BSA
PIC surface regeneration step	PIC surface regeneration and release of captured antigens	5 min	50 mM glycine + 50% *v*/*v* ethylene glycol, pH = 3	50 mM glycine + 50% *v*/*v* ethylene glycol, pH = 3
Final washing step	BSA was excluded from the washing buffer to prevent protein accumulation in the microfluidic channels of the sensors	5 min	PBS + 0.05% *v*/*v* Tween 20, pH = 7.4	MES 0.1 M, pH = 6

**Table 3:** The screening results for ASFV and CSFV obtained with the novel POC device versus the PCR results.

		ASF Sample Status		
		Positives	Negatives	Total
Screening results obtained with the POC device	Positives	122 (TP)	3 (FP)	125
	Negatives	29 (FN)	23 (TN)	52
	Total	151	26	177
		**CSF Sample Status**		
		79 (TP)	36 (FP)	115
Screening results obtained with the POC device	Positives	21 (FN)	136 (TN)	157
	Negatives	100	172	272
	Total	151	26	177

**Table 4:** Performance metrics of the novel POC device for ASF- and CSF-functionalized sensors.

	ASFV		CSFV	
Performance Metrics	Value	95% CI	Value	95% CI
Sensitivity	80.79%	73.60–86.74%	79.00%	69.71–86.51%
Specificity	88.46%	69.85–97.55%	79.07%	72.22–84.89%
Accuracy ^1^	81.92%	75.45–87.29%	79.04%	73.72–83.72%
Precision ^1^	97.60%	93.33–99.16%	68.70%	61.74–74.90%
PLR	7.00	2.41–20.36	3.77	2.78–5.13
NLR	0.22	0.15–0.31	0.27	0.18–0.39
DOR	32.25	13.63–50.87	14.21	10.17–18.28

^1^ Accuracy and precision values are affected by disease prevalence.

## Data Availability

The data presented in this study are available within the article. Raw data supporting this study are available from the corresponding author upon reasonable request.
